# Prognostic Value of Pulmonary Hypertension as an Incidental Finding Detected by Echocardiography in Patients Without Known Cardiovascular or Pulmonary Diseases

**DOI:** 10.3390/jcm14145044

**Published:** 2025-07-16

**Authors:** Avia Ashur, Amalia Levy, Noah Liel-Cohen, Ruslan Sergienko, Sergio L. Kobal

**Affiliations:** 1 Faculty of Health Sciences, Ben-Gurion University of the Negev, Beer Sheva 8410501, Israel; aviaash@clalit.org.il (A.A.); lielnoah@bgu.ac.il (N.L.-C.); sergienk@bgu.ac.il (R.S.); 2 Soroka University Medical Center, Beer Sheva 8410501, Israel

**Keywords:** echocardiography, incidental finding, mortality, prognosis, pulmonary hypertension

## Abstract

**Aims**: The global prevalence of pulmonary hypertension (PHT) among the elderly population aged 65 years and above is estimated to be 10%. While it is known to be associated with poor prognoses in patients with cardiovascular or pulmonary diseases, the significance of PHT as an incidental finding among individuals without these conditions remains unclear. The aim of this study was to investigate the relationship between incidental PHT detected by echocardiography and long-term all-cause mortality in patients without known cardiovascular or pulmonary diseases. **Methods and Results**: This retrospective, single-center cohort study included 8283 patients who underwent two consecutive echocardiographic examinations evaluating pulmonary pressure by assessing the maximal velocity of the tricuspid regurgitation jet. In total, 1705 (20.6%) patients were found to have PHT during the first echocardiography. Using a Cox proportional hazard model for all-cause mortality, PHT was found to be a significant and independent risk factor for all-cause mortality, increasing the risk by 34% (Adj. HR—1.34, 95% CI 1.21–1.47, *p* < 0.001). There was a direct relationship between PHT severity and long-term all-cause mortality, with patients with severe PHT having a two-fold higher risk compared to those with normal pulmonary blood pressure (Adj. HR—2, 95% CI: 1.58–2.54, *p* < 0.001). A “cutoff point” of sPAP > 40 mmHg was established, where pulmonary pressure values remained high and even worsened over time (*p* < 0.001). **Conclusions**: The incidental diagnosis of PHT by echocardiography in patients without known cardiovascular or pulmonary diseases is an independent risk factor for long-term all-cause mortality. Patients with sPAP ≥ 40 mmHg warrant a comprehensive clinical assessment.

## 1. Introduction

Pulmonary hypertension (PHT) is a hemodynamic state and pathophysiological disorder defined as a resting mean pulmonary artery pressure (mPAP) of ≥20 mmHg, as measured by right heart catheterization [1,2,3]. A wide range of underlying etiologies can lead to the development of PHT, including cardiac, pulmonary, and systemic disorders [4,5,6]. When present, PHT may complicate the course of many cardiovascular and pulmonary diseases, and, if left untreated, carries high morbidity and mortality rates. Importantly, several effective therapies are now available for specific forms of PHT, emphasizing the need for early recognition and diagnosis [7,8].

The estimated global prevalence of PHT is about 1%, rising to over 10% among patients older than 65 years. These percentages are expected to increase with global aging trends [9,10,11].

However, PHT diagnosis is considered a clinical challenge, as the symptoms are non-specific and may include exertional dyspnea, fatigue, weakness, or chest pain. Thus, there may be a delay of months and even years from a symptom’s onset to diagnosis. The diagnosis itself requires clinical suspicion based on symptoms, physical examination, and imaging tests [9,12]. Right heart catheterization is the definitive test for PHT diagnosis, and its performance is considered mandatory to confirm the initial clinical suspicion. However, this is an invasive test and its feasibility is limited [13,14,15,16]. In clinical practice, echocardiography is the most commonly used non-invasive tool for estimating the probability of PHT. This is typically achieved by assessing systolic pulmonary artery pressure (sPAP), calculated using the modified Bernoulli equation based on tricuspid regurgitation velocity (TRV), which provides a practical and noninvasive estimation of mPAP with good correlation to invasive measurements [1,3,12].

While PHT is a well-established marker of poor prognoses in patients with overt cardiovascular or pulmonary disease, its prognostic significance when incidentally detected in individuals without known cardiopulmonary conditions typically leading to pulmonary hypertension remains unclear. Recent data suggest that even mild elevations in pulmonary pressure, frequently overlooked in clinical practice, may have independent prognostic implications [17,18].

In this study, we aimed to investigate the association between incidentally detected PHT—identified through routine echocardiography—and long-term all-cause mortality in patients without previously diagnosed heart failure or pulmonary disease.

## 2. Materials and Methods

### 2.1. Study Population

This retrospective cohort study included 8283 patients aged 18 years or older who underwent two consecutive echocardiographic examinations at Soroka University Medical Center, a tertiary-care hospital. The first echocardiogram was performed between 2006 and 2009, and the follow-up study was conducted prior to January 2020. The median follow-up time of this analysis was 149 ± 13.77 months. Exclusion criteria included patients with at least moderate left ventricular (LV) dysfunction or significant valvular disease—defined as moderate or greater mitral or aortic stenosis or regurgitation—on the initial echocardiographic evaluation. Patients with a documented diagnosis of chronic pulmonary disease, including asthma, COPD, or congenital pulmonary disorders, as well as those with congenital heart disease at the time of the first echocardiographic study, were also excluded.

In addition, patients with other chronic diseases associated with pulmonary hypertension, diagnosed prior to the initial echocardiographic study—such as interstitial lung disease, pulmonary fibrosis, congenital lung diseases, sickle cell anemia, sarcoidosis, human immunodeficiency virus (HIV) infection, or portal hypertension—were excluded.

Further exclusions comprised patients with missing clinical or echocardiographic data and individuals who were not insured by the ‘Clalit’ Health Maintenance Organization, as their long-term outcome data could not be reliably obtained.

### 2.2. Echocardiography Study

All echocardiograms were performed according to the American Society of Echocardiography guidelines by an experienced sonographer using a General Electric systems (VIVID Q, S6 or Vivid 7, GE Vingmed Ultrasound AS, Horten, Norway) or a Philips system (Philips Medical Systems, Andover, MA, USA), and interpreted by a senior cardiologist with expertise in echocardiography. sPAP evaluation was estimated by using the modified Bernoulli equation $\mathrm{sPAP}=4\times {\mathrm{TRV}}^{2}+\mathrm{RAP}$, where TRV represents the peak velocity of tricuspid regurgitation and RAP is the right atrial pressure. RAP was assumed to be 5 mmHg, in accordance with standard echocardiographic practice when IVC assessment was not available. PHT was defined as sPAP ≥ 40 mmHg [13,19].

### 2.3. Data Collection

Data were collected from patients’ electronic medical records, including age, sex, socioeconomic index, and diagnosis of relevant chronic illness. Mortality status was determined through linkage with national death registries and Clalit HMO databases. The echocardiographic reports of all patients were reviewed for the following findings: estimated sPAP, LV systolic function, and the presence of valvular disease.

### 2.4. Statistical Analysis

The baseline characteristics of the study population were summarized using descriptive statistics. Continuous variables were compared using the *t*-test, and the chi-square test was used for categorical variables. Results are presented as the means ± SDs (standard deviations) for continuous variables. The paired *t*-test, Wilcoxon test, and McNemar test were used to evaluate the trend of change in pulmonary pressure between 2 consecutive echocardiograms. To evaluate the risk for mortality over time, a survival analysis was performed, using Kaplan–Meier analysis and Cox regression. For all analyses, a 2-sided *p* < 0.05 was considered statistically significant. Statistical analyses were conducted using SPSS software (ver. 26.0 for Windows; SPSS Inc., Chicago, IL, USA).

The study was approved by the Soroka University Medical Center IRB committee, authorization number: 0200-19-SOR.

## 3. Results

### 3.1. Study Population

**Figure 1:**
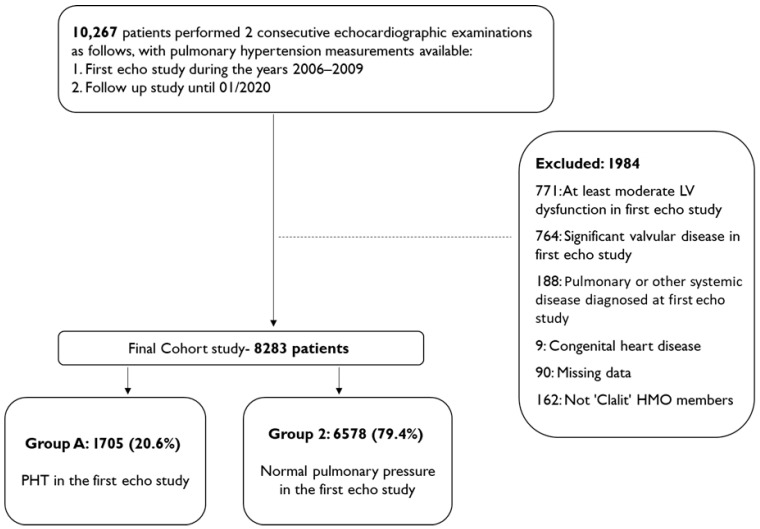
Flowchart summarizing study design, population selection and exclusion.

This study included 8283 patients who fulfilled the inclusion criteria (Figure 1).

The study population consists mainly of women (57.7%) with an average age of 63.4 ± 14.2. A total of 6594 (79.4%) patients underwent the echocardiographic study in an ambulatory setting and 1576 (18.9%) during hospitalization; the setting was unknown for 113 patients.

In total, 1705 (20.6%) patients were diagnosed with PHT in the first echocardiogram (group 1) and 6578 (79.4%) patients had normal pulmonary pressure (group 2). Patients with PHT were mostly women (64.9%) and markedly older than those with normal pulmonary pressure, with mean ages of 69.5 ± 11.4 and 61.8 ± 14.4, respectively (*p* < 0.001). The prevalence of chronic diseases and related comorbidities in each study group reflected the high overall prevalence of chronic diseases in this study cohort; however, the morbidity rate of chronic illness was consistently higher in those who were diagnosed with PHT in comparison to patients with normal pulmonary pressure (Table 1).

### 3.2. Echocardiograms

**Figure 2:**
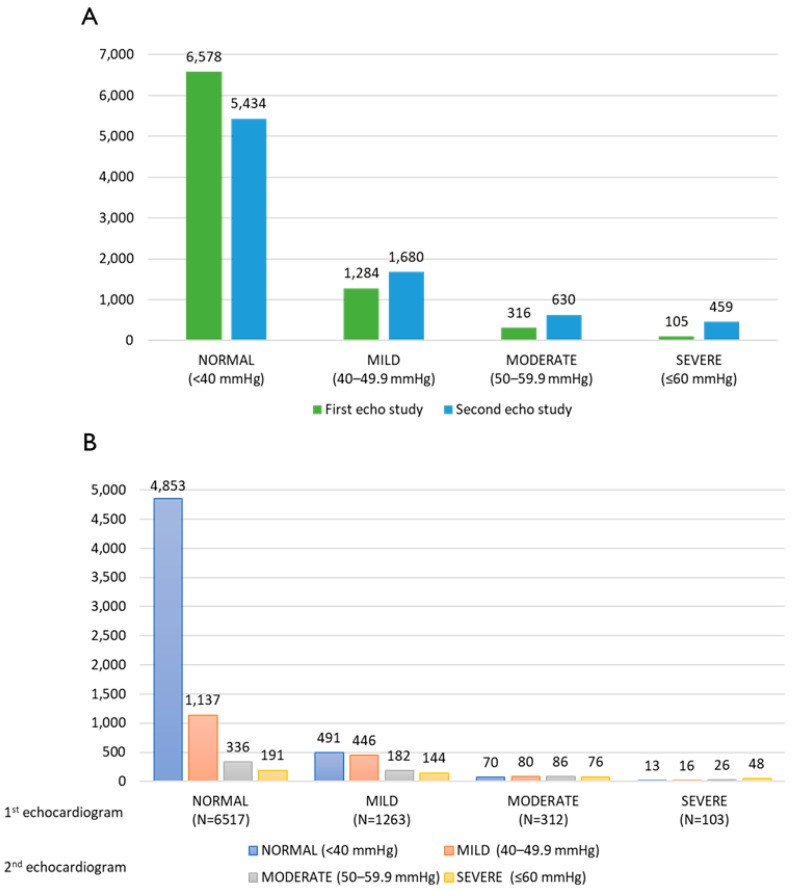
(**A**) Frequency distribution of pulmonary pressure within the cohort. (**B**) Frequency distribution of pulmonary pressure in the second echocardiogram according to the first echocardiogram results. 1st echocardiogram—N = 8283, 2nd echocardiogram—N = 8203. 80 were excluded due to missing pulmonary pressure estimates in the second study.

The frequency distribution of pulmonary pressure in both the first and second echocardiography studies is presented in Figure 2A. A significant difference was found between the pulmonary pressure values measured in two consecutive echocardiograms for the whole cohort, as the mean value in the second echocardiogram (38.27 ± 11.68 mmHg) was higher than that in the first echocardiogram (35.09 ± 7.75 mmHg) (*p* < 0.001), although still within normal limits. However, as depicted in Figure 2B, the essential details are presented: the frequency distribution of pulmonary pressure in the second echocardiography study based on the results of the first echocardiography. Among patients who had normal pulmonary pressure (sPAP < 40 mmHg) in the first echocardiography, no significant difference was observed in pulmonary pressure values measured in the subsequent echocardiography. In contrast, among patients who had PHT in the first echocardiography (sPAP > 40 mmHg), a significant difference was found in the pressure values measured in the follow-up examination (*p* < 0.001).

### 3.3. Survival Analysis

**Figure 3:**
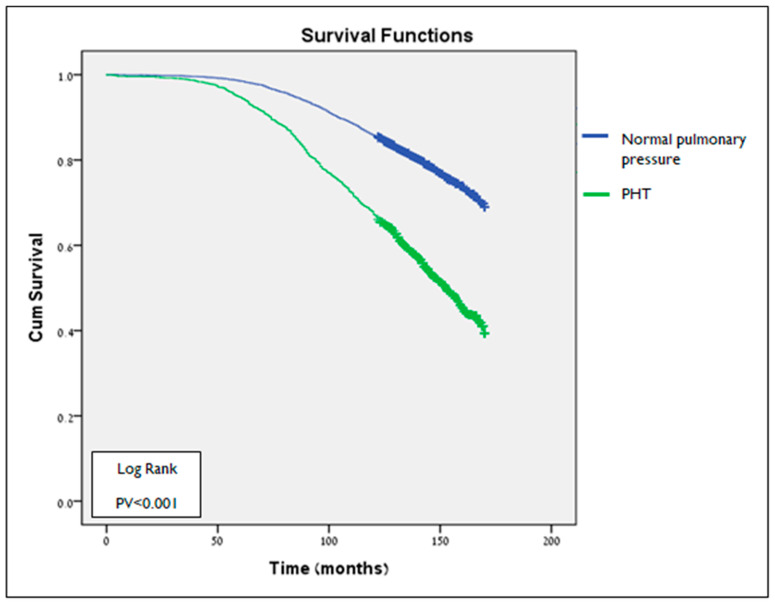
Kaplan-Meier survival plot of survival as function of time (months) in groups defined by PHT status in 1st Echo study (log-Rank test PV < 0.001). PHT—pulmonary hypertension.

The median follow-up time of this analysis was 149 ± 13.77 months. The follow-up of 1705 patients with a PHT diagnosis at the first echocardiogram prior to 01\2020 showed that 833 (48.86%) had died. Their average survival duration was 126 months. In contrast, 1513 (23%) of the 6578 patients with normal pulmonary pressure had died during this time. Their average survival duration was 140 months. These results indicate a significant difference in survival time between the two groups, as patients with PTH had a shorter survival time (*p* < 0.001) (Figure 3).

**Figure 4:**
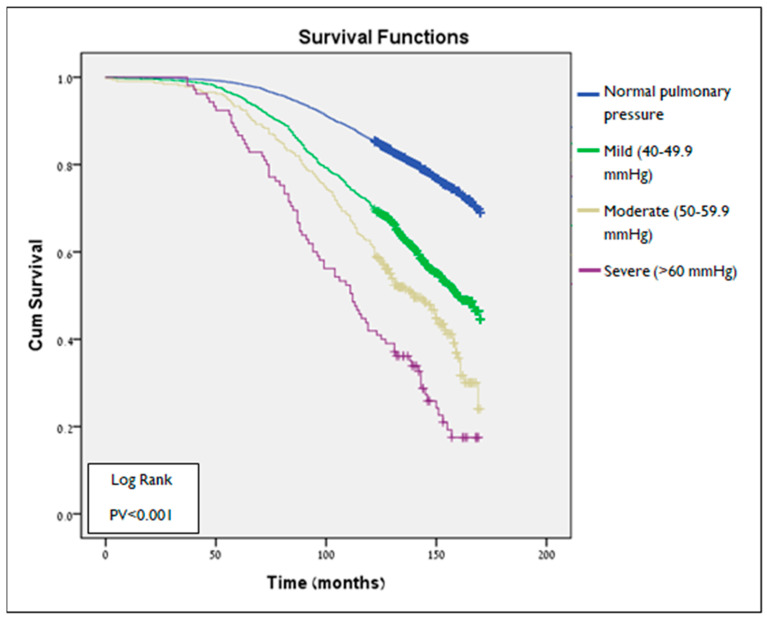
Kaplan-Meier survival plot of survival as function of time (months) in subgroups defined by PHT severity in 1st Echo study (log-Rank test PV < 0.001). PHT—pulmonary hypertension.

Additional analysis (Figure 4) compared survival time according to PHT severity degree in the first echocardiogram. Survival time was found to be significantly different among all groups (*p* < 0.001 for all comparisons). Patients with severe PHT (>60 mmHg) had the shortest mean survival time (109 ± 37 months) compared to any other group (no PHT—139 ± 25 months, mild (40–49.9 mmHg)—129 ± 33 months, and moderate (50–59.9 mmHg)—120 ± 35 months) (Figure 2B).

Table 2 shows a Cox proportional hazard model for long-term all-cause mortality. This analysis suggested that PHT is a significant and independent risk factor for all-cause mortality, with increased risk up to 34% (Adj. HR—1.34, 95% CI 1.21–1.47, *p* < 0.001). To calculate the mortality risk according to the severity of PHT, we used another Cox proportional hazard model, similar to the one presented in Table 2, apart from PHT, which is presented as an ordinal variable (Table 3). This analysis demonstrated a positive association between PHT severity and long-term all-cause mortality. Compared to patients with normal pulmonary pressure (reference group), patients with mild and moderate PHT are at increased risk by up to 27% (Adj. HR—1.27, 95% CI 1.14–1.41, *p* < 0.001) and 40% (Adj. HR—1.4, 95% CI 1.18–1.66, *p* < 0.001), respectively. Patients with severe PHT have the highest risk for all-cause mortality, as far as two times higher in patients with severe PHT compared to those with normal pulmonary blood pressure (adjusted HR—2; 95% CI: 1.58–2.54, *p* < 0.001).

## 4. Discussion

In the present study, we examined the association between incidentally detected PHT, as identified by echocardiography, and long-term all-cause mortality in patients without previously known heart failure or pulmonary disease. After adjusting for relevant demographic and clinical variables, we found that PHT was independently associated with an increased risk of long-term mortality (adjusted HR: 1.34; 95% CI: 1.21–1.47; *p* < 0.001).

Furthermore, our findings demonstrate a clear, stepwise relationship between the severity of PHT and mortality risk. Compared with patients who had normal pulmonary artery pressure, those with mild, moderate, and severe PHT exhibited significantly higher risks of death, with adjusted hazard ratios of 1.27 (95% CI: 1.14–1.41), 1.40 (95% CI: 1.18–1.66), and 2.00 (95% CI: 1.58–2.54), respectively (*p* < 0.001 for all comparisons). These results highlight the prognostic value of elevated pulmonary artery pressure as assessed non-invasively by echocardiography, even when incidentally identified.

In particular, our findings emphasize the clinical significance of TRV, a simple and widely available echocardiographic parameter. Even mildly elevated TRV—often considered incidental in low-risk individuals—was independently associated with long-term mortality, highlighting its utility as a prognostic marker even in the absence of overt cardiopulmonary disease.

Our findings are consistent with those of Stewart et al. [18], who reported a stepwise increase in all-cause mortality risk with rising levels of estimated right ventricular systolic pressure (eRVSP), with adjusted hazard ratios of 1.30, 1.82, and 2.11 for mild, moderate, and severe elevations, respectively, compared to normal eRVSP (*p* < 0.001 for all comparisons). These results closely align with our own, further supporting the prognostic value of incidentally detected pulmonary hypertension on echocardiography.

Similarly, Huston et al. [17] demonstrated that even mild echocardiographic pulmonary hypertension was independently associated with an increased risk of all-cause mortality (adjusted HR: 1.65, 95% CI: 1.46–1.86), after adjusting for key comorbidities including heart failure and left-sided valvular disease. Importantly, their findings—like ours—support the notion that the excess mortality risk associated with mild PHT is not solely explained by coexisting disease burden but rather indicates that elevated pulmonary pressure carries intrinsic prognostic significance.

Although all three studies included similarly aged populations and demonstrated comparable mortality risk ratios, several methodological differences should be noted. First, the definition of PHT differed between the studies: we defined PHT as sPAP > 40 mmHg, while Huston et al. used a threshold of eRVSP > 33 mmHg, and Stewart et al. used an even lower threshold of eRVSP > 30 mmHg. We believe that our definition may offer greater accuracy, as it incorporates RAP estimated from IVC size and collapsibility—parameters that vary with a patient’s intravascular volume status at the time of echocardiographic assessment.

Second, our study population was more selectively defined. While Stewart et al. excluded only patients with echocardiographic evidence of left heart disease, and Huston et al. included all patients referred for echocardiographic examination with complete echo data, we excluded individuals with both significant cardiac and pulmonary comorbidities. This approach enabled a clearer assessment of the prognostic impact of PHT in a relatively healthy cohort.

Finally, although all three studies included long-term follow-ups, the median duration in our study (~12 years) was longer than that of Stewart et al. (8.1 years), allowing for a more extended evaluation of long-term outcomes.

Older age and a high prevalence of chronic diseases are prominent features of this study population, especially among those suffering from PHT. We found that patients with PHT are significantly older than patients with normal pulmonary pressure (mean age of 69 ± 11.4 and 62 ± 14.4, respectively; *p* < 0.001). Furthermore, we demonstrated that patients with PHT had a high rate of relevant comorbidities, such as 88% suffering from systemic hypertension and 46% having diabetes. Despite the similar age distributions observed in the study populations of Stewart et al. and Huston et al. [17,18], as previously noted in this section, several additional studies support the demographic characteristics observed in our cohort. For instance, a large-scale study by Trammell et al., which included 110,564 patients, reported a median age of 70 years at the time of pulmonary hypertension diagnosis [20]. Similarly, Hoeper et al. noted in a 2016 review that pulmonary hypertension is frequently diagnosed in individuals over the age of 65 years [11]. Lang et al. found that 90% of patients with PHT also had systemic hypertension and 48% had diabetes, similar to our findings [21].

In our study, the prevalence of echocardiographic PHT was markedly high, up to 20%. Our results do not align with the previously published prevalence rate, which reported an estimated overall prevalence of ~1% in the global population, increasing to ~10% among patients older than 65 [10,22]. The high prevalence rate observed in our study may be secondary to the growing use of echocardiography in the global population, particularly in elderly patients. Another explanation is the composition of the study population, which is a combination of hospitalized and ambulatory patients referred to a tertiary center.

As part of our study, we evaluated the trend in systolic pulmonary artery pressure (sPAP) over time using two consecutive echocardiographic evaluations. We observed a significant increase in sPAP between the first and second echocardiograms (mean sPAP of 35.09 mmHg and 38.27 mmHg, respectively; *p* < 0.001). This trend was particularly evident in patients with an initial sPAP > 40 mmHg, in whom pulmonary pressure values remained elevated and even worsened over time (*p* < 0.001), whereas no significant change was observed in patients with initially normal sPAP.

These findings are consistent with those of Stewart et al. [18], who similarly demonstrated a progressive increase in estimated right ventricular systolic pressure (eRVSP) over time among patients who developed pulmonary hypertension, especially in those who ultimately presented with severe disease. In contrast, patients with persistently normal pulmonary pressures exhibited only negligible changes over time.

Furthermore, a recent meta-analysis by Kolte et al. [23], which included 15 studies, reported that even mild pulmonary hypertension—diagnosed via right heart catheterization or echocardiography—was associated with a 50% increase in mortality risk (*p* < 0.001). Taken together, these findings underscore the prognostic importance of the early detection of elevated pulmonary pressures.

This study has several limitations. Some clinical and demographic variables that may be relevant to the development or prognosis of PHT—such as ethnicity, BMI, and certain laboratory values—were not available in the dataset and were therefore not included in the analysis. The underlying etiology of PHT at the time of the initial echocardiographic evaluation was not known. In addition, the clinical indications for performing the echocardiographic examinations were not consistently documented, limiting our ability to assess the context in which these studies were obtained—an inherent limitation of retrospective research relying on existing medical records.

Assessment of diastolic function was not standardized throughout the study period due to evolving echocardiographic guidelines, and the clinical definitions of heart failure, particularly HFpEF, changed during this time, potentially affecting classification. Complementary data such as sleep studies, pulmonary function tests, and biomarkers including BNP were also unavailable, limiting our ability to identify conditions such as OSA, COPD, or HFpEF more precisely. Furthermore, data regarding subsequent clinical management after PHT diagnosis were not consistently available, which may represent a potential source of unmeasured confounding. Finally, as this was a retrospective, single-center study, the generalizability of our findings may be limited, and future prospective multi-center studies are warranted.

## 5. Conclusions

Incidental discovery of PHT detected by echocardiography in patients without known cardiovascular or pulmonary disease is an independent risk factor for long-term all-cause mortality. sPAP ≥ 40 mmHg may be a sign of poor prognoses, and a comprehensive clinical assessment is warranted.

## Figures and Tables

**Table 1:** Baseline demographics and clinical characteristics according to PHT status in the first echocardiographic study.

Characteristics	All Cohort N = 8283 N (%)	PHT Status N (%)		
		PHT 1705 (20.6)	Normal Pulmonary Pressure 6578 (79.4)	*p*-Value
Age, years at first echocardiogram mean ± SD	63.41 ± 14.21	69.54 ± 11.42	61.85 ± 14.4	<0.001
Female gender	4778 (57.7)	1106 (64.9)	3672 (55.8)	<0.001
Smoking	626 (7.5)	104 (6.1)	522 (7.9)	0.01
Hypertension	5945 (71.7)	1500 (88)	4445 (67.6)	<0.001
Diabetes mellitus	2802 (33.8)	776 (45.5)	2026 (30.8)	<0.001
Dyslipidemia	4893 (59)	1177 (69)	3716 (56.5)	<0.001
Ischemic heart disease	3254 (39.3)	763 (44.8)	2491 (37.9)	<0.001

Abbreviations: PHT—pulmonary hypertension.

**Table 2:** Multivariate-adjusted hazard ratios and 95% confidence intervals (adj. HR, 95% CI) for long-term all-cause mortality.

	Adj. HR	95% CI	*p*-Value
PHT diagnosis in the first echocardiogram	1.34	1.21–1.47	<0.001
Gender	0.89 *	0.82–0.98	0.02
Age at first echocardiographic study	1.07 **	1.06–1.08	<0.001
Socioeconomic index	1.22 ***	1.11–1.33	<0.001
Smoking	1.14	1.01–1.28	0.03
Hypertension	1.20	1.03–1.39	0.02
Diabetes mellitus	1.43	1.30–1.56	<0.001
Dyslipidemia	1.03	0.92–1.15	0.57
Ischemic heart disease	1.12	1.01–1.23	0.03
Event of PE\DVT in the past	1.49	1.25–1.77	<0.001
Asthma ^#^	1.06	0.91–1.24	0.48
COPD ^#^	0.93	0.8–1.08	0.36
OSA ^#^	0.89	0.75–1.06	0.19
Other chronic disease ****^,#^	1.77	1.60–1.95	<0.001
Clinical heart failure ^#^	1.40	1.26–1.55	<0.001

* Adj. HR for females compared to males. ** Adj. HR for an increase of one year in age. *** Adj. HR for a decrease of one unit in the socioeconomic index. **** Other chronic diseases: interstitial lung disease, congenital lung diseases, pulmonary fibrosis, sickle cell anemia, sarcoidosis, HIV, and portal hypertension. # Diagnosis time post-echocardiogram 1.

**Table 3:** Multivariate-adjusted hazard ratios and 95% confidence intervals (adj. HR, 95% CI) for long-term all-cause mortality by pulmonary hypertension severity.

	Adj. HR	95% CI	*p*-Value
Pulmonary pressure in the first echocardiogram			
Normal	Refence Group		
Mild PHT	1.27	1.14–1.41	<0.001
Moderate PHT	1.40	1.18–1.66	<0.001
Severe PHT	2.00	1.58–2.54	<0.001
Gender	0.89 *	0.81–0.97	0.12
Age at first echocardiographic study	1.07 **	1.06–1.08	<0.001
Socioeconomic index	1.21 ***	1.11–1.33	<0.001
Smoking	1.17	0.99–1.38	0.05
Hypertension	1.19	1.03–1.39	0.02
Diabetes mellitus	1.42	1.29–1.55	<0.001
Dyslipidemia	1.04	0.93–1.16	0.47
Ischemic heart disease	1.11	1.01–1.23	0.04
Event of PE\DVT in the past	1.49	1.25–1.77	<0.001
Asthma ^#^	1.07	0.92–1.26	0.36
COPD ^#^	0.94	0.81–1.11	0.46
OSA ^#^	0.88	0.74–1.04	0.12
Other chronic disease ****^,#^	1.73	1.57–1.92	<0.001
Clinical heart failure ^#^	1.38	1.24–1.53	<0.001
LV systolic dysfunction in second echocardiogram			
Normal LV function	Refence Group		
Mild	1.23	1.08–1.41	0.003
Mild–moderate to moderate	1.24	1.08–1.42	0.002
Moderate–severe to severe	1.59	1.38–1.83	<0.001

* Adj. HR for females compared to males. ** Adj. HR for an increase of one year in age. *** Adj. HR for a decrease of one unit in the socioeconomic index. **** Other chronic diseases: interstitial lung disease, congenital lung diseases, pulmonary fibrosis, sickle cell anemia, sarcoidosis, HIV, and portal hypertension. # Diagnosis time post-echocardiogram 1.

## Data Availability

The data that support the findings of this study are available from the corresponding author upon reasonable request.
